# Procalcitonin Impairs Liver Cell Viability and Function In Vitro: A Potential New Mechanism of Liver Dysfunction and Failure during Sepsis?

**DOI:** 10.1155/2017/6130725

**Published:** 2017-02-01

**Authors:** Martin Sauer, Sandra Doß, Johannes Ehler, Thomas Mencke, Nana-Maria Wagner

**Affiliations:** ^1^Clinic for Anesthesiology and Intensive Care Medicine, University Hospital Rostock, Rostock, Germany; ^2^Fraunhofer Institute for Cell Therapy and Immunology, EXIM, Rostock, Germany

## Abstract

*Purpose*. Liver dysfunction and failure are severe complications of sepsis and result in poor outcome and increased mortality. The underlying pathologic mechanisms of hepatocyte dysfunction and necrosis during sepsis are only incompletely understood. Here, we investigated whether procalcitonin, a biomarker of sepsis, modulates liver cell function and viability.* Materials and Methods*. Employing a previously characterized and patented biosensor system evaluating hepatocyte toxicity in vitro, human hepatocellular carcinoma cells (HepG2/C3A) were exposed to 0.01–50 ng/mL procalcitonin for 2 × 72 h and evaluated for proliferation, necrosis, metabolic activity, cellular integrity, microalbumin synthesis, and detoxification capacity. Acetaminophen served as positive control. For further standardization, procalcitonin effects were confirmed in a cellular toxicology assay panel employing L929 fibroblasts. Data were analyzed using ANOVA/Tukey's test.* Results*. Already at concentrations as low as 0.25 ng/mL, procalcitonin induced HepG2/C3A necrosis (*P* < 0.05) and reduced metabolic activity, cellular integrity, synthesis, and detoxification capacity (all *P* < 0.001). Comparable effects were obtained employing L929 fibroblasts.* Conclusion*. We provide evidence for procalcitonin to directly impair function and viability of human hepatocytes and exert general cytotoxicity in vitro. Therapeutical targeting of procalcitonin could thus display a novel approach to reduce incidence of liver dysfunction and failure during sepsis and lower morbidity and mortality of septic patients.

## 1. Introduction

Sepsis is one of the leading causes of death in critically ill patients admitted to intensive care units [1]. High mortality rates are due to the development of multiple organ dysfunction syndrome (MODS) [2]. Hepatic dysfunction is a key component of MODS and liver failure occurs in nearly 19% of patients with septic shock. Hepatic dysfunction thereby crucially contributes to poor prognosis and outcome [3]. In the pathophysiology of sepsis, the liver modulates several aspects of disease initiation and progression. For example, it clears bacteria and endotoxin from the splanchnic area and tailors host immune responses by secreting inflammatory mediators [4, 5]. Although impairment of liver cell function is thus one of the most crucial amplifiers of MODS during sepsis, its pathophysiology during sepsis is still incompletely understood [6].

Procalcitonin is considered one of the earliest and most predictive biomarkers for the identification, treatment, and prognosis of septic patients. Procalcitonin is a 116-amino acid precursor protein of calcitonin secreted by endotoxin- or pathogen-activated monocytes [7, 8]. During the course of bacterial sepsis, further procalcitonin secretion by parenchymal cells of various organs results in a burst of procalcitonin plasma concentrations of up to 100,000-fold [7, 9, 10]. In animal models, procalcitonin administration to septic hamsters results in doubled lethality rates [11]. Conversely, procalcitonin neutralization during sepsis improves survival in hamsters and pigs [11, 12], suggesting procalcitonin* itself* to play a role in sepsis pathology [13]. In this regard, we have recently identified procalcitonin to impair endothelial cell viability and function and thus to potentially contribute to disturbed microcirculation and hypoperfusion during sepsis [14].

In the present study, we aimed to elucidate a potential direct role of procalcitonin in modulating liver cell function. We employed a previously patented [15] and characterized biosensor system [16] investigating the effects of 0.01–50 ng/mL procalcitonin on vitality, viability, and function of hepatocytes. We further verified results in a standardized toxicology screening assay employing murine fibroblasts to further characterize procalcitonin cytotoxic potency in vitro.

## 2. Methods

### 2.1. Cell Culture, Cell Proliferation, and Vitality

A human hepatocyte cell line HepG2/C3A was obtained from the American Type Culture Collection (ATCC CRL-10741, WCB 25022009) and cultivated in Dulbecco's modified Eagle's Medium (Dulbecco's DMEM, GIBCO Life Technologies, Eggenstein, Germany), supplemented with 10% fetal calf serum (PAA Laboratories, Cölbe, Germany), 1% of glutamine solution (PAA), and 1% of antibiotics solution (Penicillin G: 10,000 IE/mL/Streptomycin: 10 mg/mL; PAA). For incubation with procalcitonin, 250,000 hepatocytes were seeded per well of a 24-well plate, incubated in 1 mL medium for 72 h, and exposed to procalcitonin (Sigma-Aldrich, Steinheim am Albuch, Germany) ranging from 0.01 to 50 ng/mL or phosphate buffered saline (PBS) only serving as vehicle control (negative control). Medium was then removed and replaced by fresh medium supplemented with similar procalcitonin concentrations or PBS. Following 72 h, proliferation and vitality were assessed by trypan blue staining technique and manual cell counts. Exposure to acetaminophen (15.24 mM, Sigma-Aldrich) for two times 72 h served as positive control [15].

### 2.2. Cell Viability

Hepatocyte viability was investigated following incubation of cells with 2,3-bis(2-methoxy-4-nitro-5-sulfophenyl)-5-[(phenylamino)carbonyl]-2H-tetrazolium hydroxide (XTT) which is metabolically reduced in viable cells by mitochondrial dehydrogenase to a water-soluble formazan product detectable by absorbance readings [16, 17]. LDH determination from cell culture medium supernatant was performed according to the optimized standard method of the German Society of Clinical Chemistry (Deutsche Gesellschaft für Klinische Chemie, DGKC). Pyruvate was employed as a substrate and NADH decrease was determined photometrically (Cobas Mira, Roche) [18].

### 2.3. Hepatocyte Function

For the determination of microalbumin synthesis, hepatocytes were incubated with procalcitonin for two times 72 h and supernatants were collected, centrifuged, and stored at −80°C pending analysis. Microalbumin was then determined nephelometrically (Immage 800, Beckmann Coulter GmbH, Krefeld, Germany) [19]. Induction of hepatocyte cytochrome 1A2 activity was evaluated by incubation of hepatocytes with 7-ethoxyresorufin (7-ER), a fluorimetric substrate and suicide inhibitor of cytochrome P450 reflecting the activity of cytochrome 1A2 by ethoxyresorufin-O-deethylase (EROD) activity [20].

### 2.4. Murine Fibroblasts Toxicology Screen

Murine fibroblasts were grown in Dulbecco's modified Eagle's Medium supplemented with 5% fetal calf serum, 1% of glutamine solution (PAA), and 1% of antibiotics solution according to 9935 ISO Norm. Effects of 0.01–100 ng/mL procalcitonin on proliferation, vitality, and mitochondrial dehydrogenase were investigated as mentioned above.

### 2.5. Statistical Analysis

Data was analyzed employing GraphPad Prism Software version 7.01 (GraphPad Prism Software Inc., La Jolla, CA, USA) using One-Way ANOVA followed by Tukey's test for correction of multiple comparisons. The results are expressed as mean and standard deviation (SD). Differences were considered significant at a *P* value of ≤0.05.

## 3. Results

### 3.1. Procalcitonin Impairs Hepatocyte Vitality and Viability

**Figure 1:**
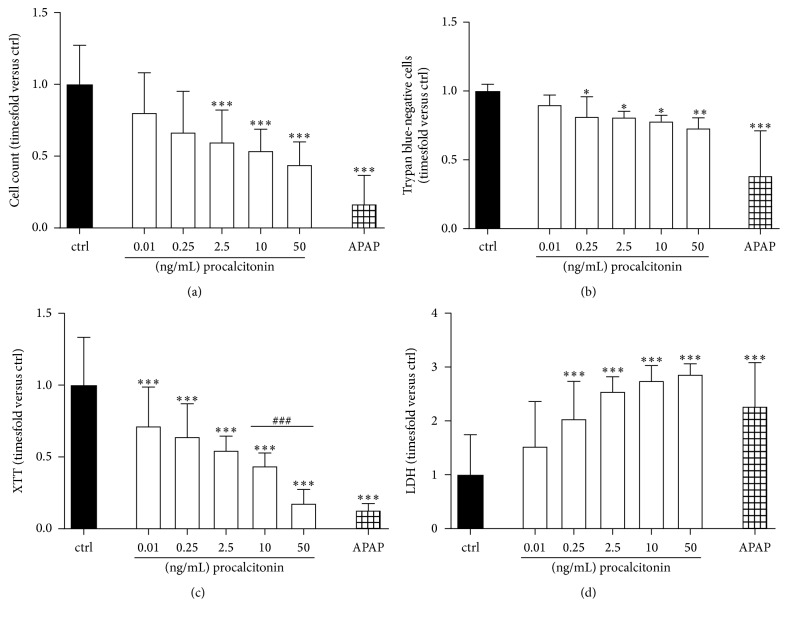
(a) Cell counts, (b) number of trypan blue-negative cells, (c) XTT metabolization, and (d) LDH release of HepG2/C3A cells following exposure to procalcitonin for 2 × 72 h in concentrations as indicated. APAP acetaminophen 15.24 mM. *n* = 18–28 biological replicates; ^*∗*^*P* < 0.05 and ^*∗∗∗*^*P* < 0.001 versus ctrl; *P* < 0.05, *P* < 0.01, and ^###^*P* < 0.001 versus as indicated. Significance between concentrations is only indicated if differences between consecutive concentrations employed reach statistical significance.

Incubation of hepatocytes with procalcitonin ranging from 0.01 to 50 ng/mL dose dependently inhibited hepatocyte proliferation over the time-course of 72 h with significant effects starting at 2.5 ng/mL (0.55 ± 0.23-fold versus ctrl, *P* < 0.001; Figure 1(a)). Beginning at low concentrations as 0.25 ng/mL, procalcitonin also affected hepatocyte viability indicated by reduced numbers of trypan blue-negative cells (0.82 ± 0.15-fold versus ctrl, *P* < 0.05; Figure 1(b)). Incubation with procalcitonin at all concentrations employed severely impacted basic cell metabolism (0.72 ± 0.23-fold versus ctrl for 0.01 ng/mL, *P* < 0.001, Figure 1(c)) and concentrations starting at 0.25 ng/mL induced the release of LDH into cell culture supernatants (2.04 ± 0.07-fold versus control, *P* < 0.001, Figure 1(d)). For proliferation, vitality, and metabolism, only high concentrations of procalcitonin (i.e., 50 ng/mL) induced comparable loss of cell integrity as acetaminophen. For LDH release, already 2.5 ng/mL induced comparable effects as acetaminophen employed as positive control.

### 3.2. Procalcitonin Reduces Hepatocyte Synthesis and Detoxification Potency

**Figure 2:**
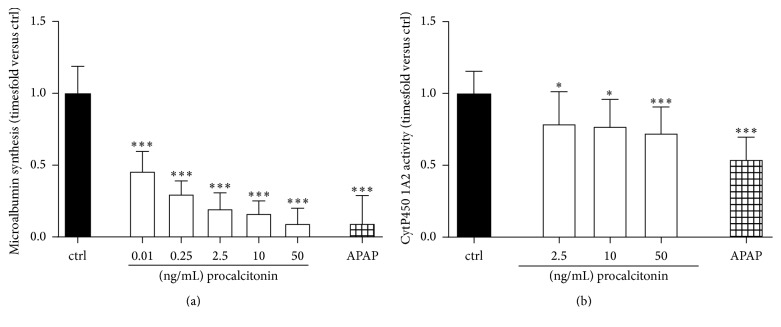
(a) Microalbumin synthesis (*n* = 9-10 biological replicates) and (b) cytochrome 1A2 detoxification (*n* = 36 biological replicates) activity of HepG2/C3A cells following exposure to procalcitonin for 2 × 72 h in concentrations as indicated. APAP acetaminophen 15.24 mM. *n* = 18–28; ^*∗*^*P* < 0.05 and ^*∗∗∗*^*P* < 0.001 versus ctrl. Significance between concentrations is only indicated if differences between consecutive concentrations employed reach statistical significance.

Next, we investigated the degree procalcitonin-mediated affection of vitality and viability has implications for hepatocyte albumin synthesis and ethanol clearance. In this regard, all concentrations of procalcitonin employed severely affected microalbumin synthesis with already low concentrations such as 0.01 ng/mL reducing synthesis capacity to only half of that of control cells (0.46 ± 0.14-fold versus ctrl for 0.01 ng/mL, *P* < 0.001, Figure 2(a)). The activity of the cytochrome P450 1A2 detoxification system was significantly affected at concentrations of 2.5 ng/mL (0.79 ± 0.23-fold versus ctrl, *P* < 0.05; Figure 2(b)).

### 3.3. Procalcitonin Exerts General Cytotoxicity

**Figure 3:**
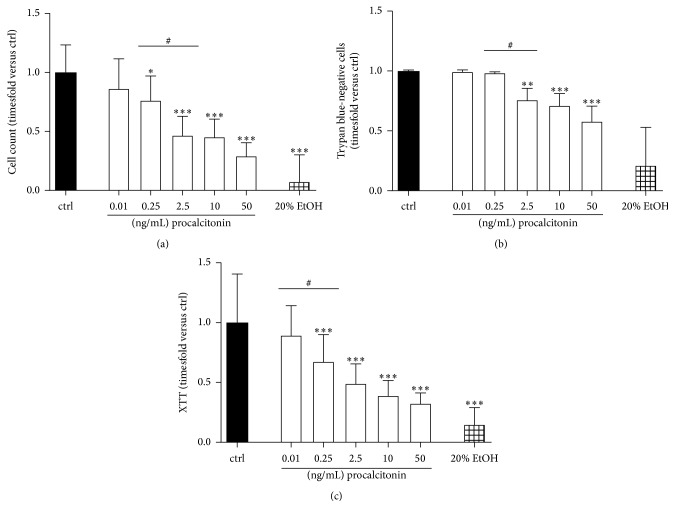
(a) Cell counts (*n* = 9–18), (b) number of trypan blue-negative cells (*n* = 9–18), and (c) XTT metabolization (*n* = 18–36 biological replicates) of L929 fibroblasts following exposure to procalcitonin for 2 × 72 h in concentrations as indicated. EtOH ethanol 20%. *n* = 18–28 biological replicates; ^*∗*^*P* < 0.05, ^*∗∗*^*P* < 0.01, and ^*∗∗∗*^*P* < 0.001 versus ctrl; ^#^*P* < 0.05 versus as indicated. Significance between concentrations is only indicated if differences between consecutive concentrations employed reach statistical significance.

Results from analyzing hepatocytes indicating cytotoxic effects of procalcitonin were further verified in a standardized cell-based toxicology assay employing L929 fibroblasts. Here, procalcitonin beginning at 0.25 ng/mL affected cell proliferation indicated by lower cell counts following 2 × 72 h of incubation (0.76 ± 0.21-fold versus ctrl, *P* < 0.05, Figure 3(a)). Beginning at similar concentrations, procalcitonin reduced cellular viability (reduction to 0.82 ± 0.25-fold trypan blue-negative cells compared to untreated control, *P* < 0.05, Figure 3(b)). Again starting at low 0.25 ng/mL, procalcitonin significantly reduced basal cell metabolism (to 0.68 ± 0.23-fold compared to ctrl, *P* < 0.001, Figure 3(a)).

## 4. Discussion

Hepatic function is affected already during early stages of systemic infection. One of the first signs of hepatic dysfunction is an impairment of bile production. Under experimental conditions, a reduced canalicular bile secretion can be detected within minutes after initialization of endotoxemia [21, 22]. In this regard, previous studies identified that 10.9% of patients newly admitted to ICU exhibit bilirubin serum levels >34.2 *μ*mol/L within 48 h of admission [23]. Histologic analysis of liver biopsies suggests rapid development of ubiquitous centrilobular necrosis and hepatocellular apoptosis during the course of sepsis [24]. These findings point towards a rapid onset of hepatocyte loss of integrity and functional impairment during the course of sepsis. Procalcitonin levels rise early during the course of systemic infection. While in healthy humans procalcitonin plasma concentration is mostly below 0.01 ng/mL, consideration of sepsis is recommended if procalcitonin plasma levels exceed 0.5 ng/mL [25]. In the present study, we found that procalcitonin affects hepatocyte vitality, viability, and function already during lower concentrations, mostly at 0.25 ng/mL. A dose dependent cell lysis was started even in very low concentrations of procalcitonin, displayed in high and increasing levels of lactate dehydrogenase in the cell culture supernatant. Procalcitonin similarly and mostly dose dependently affected all qualities of cellular function investigated such as proliferation, induction of cell death, metabolism, integrity, microalbumin synthesis, and detoxification capacity. Our results thus point towards procalcitonin as a potent and, importantly, early mediator of hepatic dysfunction during sepsis.

Chemokines and cytokines impair cellular functions and viability of primary hepatocytes and HepG2/C3A cells [26–29]. Proinflammatory cytokines, for instance, are known to downregulate albumin synthesis [27], to cause a dysfunction of mitochondria [26], and to diminish function of some P450 cytochromes like CYP 1A2, CYP 2E1, and CYP 7A1 [28, 29]. The panel of assays to study hepatocyte function and viability employed in this study has been previously patented for the early detection of patients endangered for the development of liver dysfunction during sepsis [15, 16]. Further standardization was done by the employment of the panel for screening purposes during extracorporeal treatments and for an experimental model of liver dysfunction in* Schistosoma* infections [30, 31]. It utilizes the human hepatocellular carcinoma cell line HepG2/C3A, a well-characterized subclone of the hepatoma-derived HepG2 cell line often used for toxicological screening [32–34]. Biosynthesis capacity of plasma proteins and inflammatory mediators and the cell surface receptors expressed are highly similar to those of normal hepatocytes (see Sauer et al. for review, [16]). In addition, cellular ability for cytochrome P450 monooxidase-mediated detoxification and glucuronic- and sulfate-conjugation are also equal to those of hepatocytes. However, this in vitro testing system does not resemble the multitude of actors and processes hepatocytes within the liver tissue are exposed to during sepsis, which is a major limitation of this study. Further investigations are thus needed to analyze whether procalcitonin* itself* is a mediator of liver cell dysfunction during sepsis.

Interestingly, procalcitonin's ability to identify bacteraemia does not apply to patients suffering from liver failure. Hepatocyte necrosis correlates with high levels of procalcitonin irrespective of infection and authors concluded that the massive inflammation associated with hepatocyte necrosis may have triggered excess procalcitonin release [35]. During early stages of proinflammatory conditions, procalcitonin is secreted by cytokine-activated macrophages during their interaction with endothelial cells [7]. With persisting presence of proinflammatory mediators in the blood, further procalcitonin production and release is initiated in the parenchyma of a variety of organs, resulting in 10,000–100,000-fold increase of procalcitonin plasma concentration [9]. The results of the present study now point towards procalcitonin* itself* to induce hepatocyte dysfunction and impairment of cellular integrity. This suggests procalcitonin as part of a vicious circle of sterile inflammation and liver cell necrosis. Future studies may thus address whether procalcitonin can be a potential target for the treatment of acute liver failure, the importance of inflammatory mediators, or whether the interplay of hepatocytes and procalcitonin is independent of infection.

Sepsis is associated with vascular barrier dysfunction, fluid extravasation, and rapid induction of microcirculatory disturbance. Our previous study identified procalcitonin to induce, within a few hours, endothelial cell dysfunction and endothelial cell death [14]. Liver failure occurs during late stages of sepsis-induced multiple organ dysfunction syndrome and hepatocytes were therefore exposed to procalcitonin for time periods of 6 days in the present study. Although times of incubation thus greatly differed, much higher concentrations of procalcitonin (i.e., 100 ng/mL for 6 h) were needed to induce endothelial cell necrosis compared to only 0.25 ng/mL procalcitonin to induce hepatocyte necrosis in the present study. In the standardized cellular toxicology assays employing L929 fibroblasts, again higher procalcitonin concentrations, that is, 2.5 ng/mL, were needed to induce cell death in a significant number of cells. Other studies employing renal mesangial cells reported similarly high concentrations of procalcitonin (i.e., 2.5–5 ng/mL) to induce cell apoptosis [36], suggesting that hepatocytes may belong to more susceptible and other cell types such as endothelial or mesangial cells to a more insensitive extreme of a continuum of procalcitonin effects on cell function and viability. A limitation of this study is that we do not provide a mechanism of procalcitonin's actions on liver cell and fibroblast function that does apply to all the observations made. However in previous studies analysis of gene expression profiles in endothelial cells exposed to procalcitonin revealed regulation of genes involved in various cellular functions such as cell metabolism and inflammatory pathways [14]. Further studies are needed to investigate the underlying effects of how procalcitonin affects cellular integrity and whether neutralization of procalcitonin would modulate the occurrence of organ dysfunction and outcome during sepsis or other entities associated with increased procalcitonin plasma concentrations.

## 5. Conclusion

In conclusion, our experiments provide evidence for procalcitonin to directly impair function and viability of human hepatocytes and exert general cytotoxicity in vitro. The mechanisms for the hepatotoxicity of procalcitonin are unclear and need further investigations in suitable in vitro and in vivo models.
